# Right atrial infarction causing recurrent cardiac rupture and cardiac tamponade

**DOI:** 10.1093/ehjcr/ytad534

**Published:** 2023-10-27

**Authors:** Ryota Kakizaki, Ryuta Imaki, Junya Ako

**Affiliations:** Department of Cardiovascular Medicine, Kitasato University School of Medicine, 1-15-1 Kitasato, Minami-ku, Sagamihara 252-0373, Japan; Department of Cardiovascular Medicine, Yamato municipal hospital, Yamato 242-8602, Japan; Department of Cardiovascular Medicine, Yamato municipal hospital, Yamato 242-8602, Japan; Department of Cardiovascular Medicine, Kitasato University School of Medicine, 1-15-1 Kitasato, Minami-ku, Sagamihara 252-0373, Japan

An 81-year-old female presented to our hospital due to chest pain. An electrocardiogram showed ST segment elevation in leads II, III, and aVF without PQ segment change. Echocardiography demonstrated hypokinetic motion in the inferior wall of the left ventricle and pericardial effusion without chamber collapse. Computed tomography (CT) before coronary angiography demonstrated calcification in the right coronary artery (RCA) and multiple nodular lesions in the adipose tissue around the right atrium (RA) (Panels A and B). Coronary angiography showed occlusion in the proximal RCA (Panel C). After administration of aspirin and prasugrel with loading dose, a drug-eluting stent was implanted in the proximal RCA. On day 3 of dual antiplatelet therapy, she suffered sudden pulseless electrical activity due to cardiac tamponade. Her spontaneous circulation returned after cardiopulmonary resuscitation and pericardial drainage of sanguineous effusion. After pericardial effusion stopped spontaneously, antithrombotic therapy resumed with aspirin and warfarin with a target international normalized ratio of 2.0–2.5 because atrial fibrillation was detected. Subsequently, cardiac tamponade with sanguineous pericardial effusion recurred on day 30. She died on day 44, and the autopsy revealed a haematoma extending from RA to adventitia adipose tissue (Panel D). Post-hemorrhagic changes, coagulation necrosis, and replacement fibrosis suggesting myocardial infarction were observed in RA myocardium (Panels E and F).

RA infarction is a rare complication of acute myocardial infarction and can cause cardiac rupture. Diagnosis of RA infarction is sometimes difficult. A haematoma in the adipose tissue around the RA and pericardial effusion demonstrated in CT may be helpful findings for diagnosing RA infarction with cardiac rupture. To avoid recurrent rupture, surgical therapy should be considered for patients with these findings.

**Figure:**
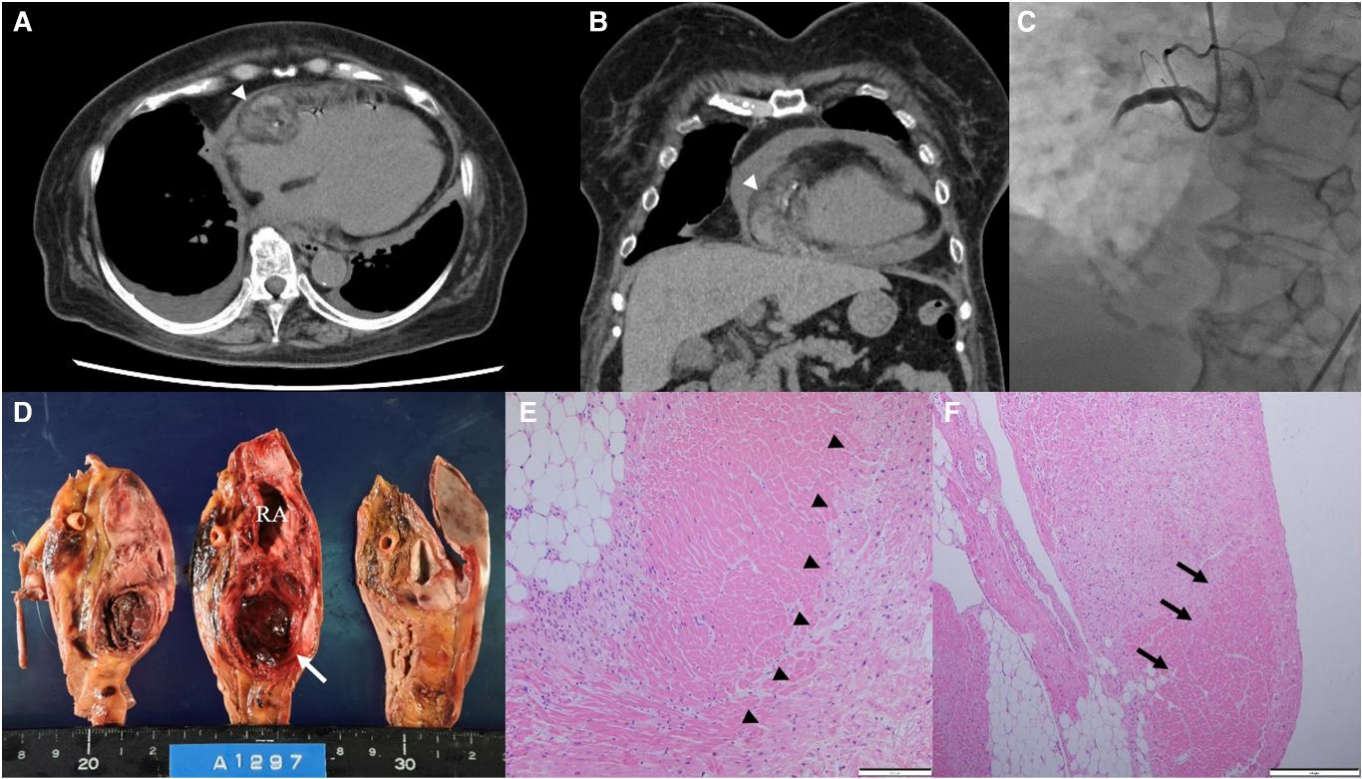


## Data Availability

The data underlying this article will be shared on reasonable request to the corresponding author.

## Notes

**Consent:** written informed consent for scientific use of the data was obtained from the patient’s daughter.

**Funding:** No funding for this article.

